# A multiscale chemo-electro-mechanical skeletal muscle model to analyze muscle contraction and force generation for different muscle fiber arrangements

**DOI:** 10.3389/fphys.2014.00498

**Published:** 2014-12-23

**Authors:** Thomas Heidlauf, Oliver Röhrle

**Affiliations:** ^1^Continuum Biomechanics and Mechanobiology Research Group, Institute of Applied Mechanics (CE), University of StuttgartStuttgart, Germany; ^2^Stuttgart Research Center for Simulation Technology (SimTech), University of StuttgartStuttgart, Germany

**Keywords:** spanning-fibered, series-fibered, sarcomere stretch, sarcomere instability, biophysical cell model, non-isometric

## Abstract

The presented chemo-electro-mechanical skeletal muscle model relies on a continuum-mechanical formulation describing the muscle's deformation and force generation on the macroscopic muscle level. Unlike other three-dimensional models, the description of the activation-induced behavior of the mechanical model is entirely based on chemo-electro-mechanical principles on the microscopic sarcomere level. Yet, the multiscale model reproduces key characteristics of skeletal muscles such as experimental force-length and force-velocity data on the macroscopic whole muscle level. The paper presents the methodological approaches required to obtain such a multiscale model, and demonstrates the feasibility of using such a model to analyze differences in the mechanical behavior of parallel-fibered muscles, in which the muscle fibers either span the entire length of the fascicles or terminate intrafascicularly. The presented results reveal that muscles, in which the fibers span the entire length of the fascicles, show lower peak forces, more dispersed twitches and fusion of twitches at lower stimulation frequencies. In detail, the model predicted twitch rise times of 38.2 and 17.2 ms for a 12 cm long muscle, in which the fibers span the entire length of the fascicles and with twelve fiber compartments in series, respectively. Further, the twelve-compartment model predicted peak twitch forces that were 19% higher than in the single-compartment model. The analysis of sarcomere lengths during fixed-end single twitch contractions at optimal length predicts rather small sarcomere length changes. The observed lengths range from 75 to 111% of the optimal sarcomere length, which corresponds to a region with maximum filament overlap. This result suggests that stability issues resulting from activation-induced stretches of non-activated sarcomeres are unlikely in muscles with passive forces appearing at short muscle length.

## 1. Introduction

The fascicles in parallel-fibered muscle are aligned with the muscle's line of action and run almost the entire length of the muscle (Loeb et al., 1987). The fascicles either consist of long fibers spanning the entire length of the fascicles (in the following termed “spanning-fibered muscle”), or they are composed of several shorter in-series arranged fiber compartments (in the following termed “series-fibered muscle”) (Richmond et al., 1985; Heron and Richmond, 1993; Young et al., 2000). The fiber compartments in series-fibered muscle can either be separated by tendinous inscriptions, as, for example, in cat and human semitendinosus muscle, or the muscle fibers are arranged in short overlapping arrays (Loeb et al., 1987; Paul, 2001; Woodley and Mercer, 2005).

The advantages and disadvantages of series-fibered and spanning-fibered muscle arrangements on the force generation have not yet been systematically analyzed. Experiments provide only limited information on which effects are due to the fiber arrangement, and which effects are due to other anatomical or physiological properties, e. g., the muscle geometry. Mathematical models instead can be used to investigate the influence of a specific property on the overall behavior. Previous modeling works focused on the influences of the muscle geometry and the fiber direction on the force generation (Zuurbier and Huijing, 1992; Sánchez et al., 2014). To investigate the effect of different fiber arrangements, one requires a model that unifies the following features: (i) The dynamics of the active force generation are determined at discrete locations (“sarcomeres”) along a muscle fiber. (ii) The model takes into account the subsequent activation of adjacent “sarcomeres” through the propagation of action potentials (APs) along the fibers. This is required since the AP propagation speed is rather slow, and hence, sarcomere activation is non-synchronized along a muscle fiber, and the asynchronism increases with increasing fiber length. (iii) The model accounts for the muscle tissue, in which the muscle fibers are embedded, and which shows resistance to applied loads. The tissue representation is required due to the fact that isolated muscle fibers or myofibrils do not behave like fibers within a muscle (Prado et al., 2005).

There exists no muscle model that can incorporate all of these requirements. Hill-type muscle models are typically used to describe whole muscle behavior (Zajac, 1989), although they have also been used to model single sarcomeres, and, by in-series arranging multiple Hill-type models, myofibrils and muscle fiber segments have been modeled (Morgan et al., 1982; Stoecker et al., 2009; Günther et al., 2012). While these approaches can describe local changes in sarcomere length, they cannot capture the behavior of a fiber within the three-dimensional (3D) muscle tissue. This is due to the fact that the passive forces in isolated myofibrils and single muscle fibers are mainly attributed to the titin filament (Horowits, 1992; Denoth et al., 2002), unlike in muscle tissue, where the extracellular matrix contributes additional passive forces (Prado et al., 2005). Further, in isolated myofibrils and single muscle fibers, force transmission can only take place along their length. In whole muscle, however, force transmission also occurs in lateral direction (Huijing, 1999). For an adequate representation of fibers within the muscle tissue and its mechanical implications on the behavior of the whole muscle, a 3D model based on continuum-mechanical principles is required.

Previous continuum-mechanical skeletal muscle models (Blemker et al., 2005; Röhrle and Pullan, 2007) include the active force-length (*F*-*ℓ*) and/or the active force-velocity (*F*-*v*) relations on the macroscale, which implies the assumption of an averaged sarcomere length and an averaged sarcomere shortening velocity. Therefore, these modeling approaches cannot represent local changes in sarcomere length and shortening velocity, which are required for above motivated cases. Furthermore, both the *F*-*ℓ* and the *F*-*v* relations should be modeled on the microscale, since they can be attributed to properties on the sarcomere level. The length dependence of the active force is due to changes in the overlap of the thick and thin filaments within the sarcomeres (Gordon et al., 1966), while the velocity dependence is attributed to (i) a lower tension of the cross bridges (XBs) that reattach in a shortened state, and (ii) an increased XB-detachment rate (Piazzesi et al., 2007; Telley and Denoth, 2007).

To overcome the limiting modeling assumption of homogenized sarcomere lengths and shortening velocities, and to analyze the effects of different fiber arrangements, in this contribution, the multiscale chemo-electro-mechanical skeletal muscle model of Heidlauf and Röhrle (2013) is extended to include the *F*-*ℓ* and *F*-*v* relations on the microscale.

## 2. Materials and methods

To model the active *F*-*ℓ* and *F*-*v* relations on the microscale, the biophysical half-sarcomere model of Shorten et al. (2007) is extended to non-isometric conditions. The extended half-sarcomere model is coupled to (i) bioelectrical field equations describing the propagation of APs along muscle fibers, and (ii) a 3D continuum-mechanical description of the muscle tissue (Heidlauf and Röhrle, 2013).

### 2.1. Detailed overview of the multiscale model

Depolarization of the membrane potential of the biophysical half-sarcomere model located at the innervation zone is induced by a current injection of short duration. The timing of the current injections is given by the stimulation frequency, which is prescribed in this study (e. g., 50 Hz or 100 Hz). The constant firing frequency can also be replaced by discrete motor unit discharge times resulting, for example, from the decomposition of an EMG signal (De Luca and Hostage, 2010) or from a phenomenological (Fuglevand et al., 1993) or biophysical (Heidlauf and Röhrle, 2013) model of the α motor neurons. Based on the respective stimulation, the biophysical half-sarcomere model provides, among many others, two quantities that are essential for the multiscale framework—the locally generated active stresses and the changes in membrane potential due to ionic and capacitive currents. To simulate the propagation of APs, the bioelectrical field equations are used to describe the diffusion of the membrane potential along the fibers. This results in a bi-directional coupling between the half-sarcomere model and the bioelectrical field equations through the membrane potential. The locally-generated, sarcomere-based active stresses are included in the formulation of the continuum-mechanical constitutive relation (relation between local deformation and resulting local stresses). The continuum-mechanical model predicts the deformation of the muscle geometry, the internal stress and strain distributions, and the forces that can be passed to adjacent structures such as tendon. The local strain is used to determine the new sarcomere length and the sarcomere shortening velocity, which are in turn inputs to the biophysical half-sarcomere model. Hence at a point in space, the half-sarcomere model and the continuum-mechanical model are bi-directionally coupled. Furthermore, since deformation changes geometrical properties of the fibres, the AP propagation along a muscle fiber is solved on the deformed geometry.

Due to the complexity of the model, Table 1 lists the model's variables including their dependencies, while Table 2 summarizes the parameters of the model.

**Table 1:** **Model variables**.

**Symbol**	**Description**
*t*	time
**X**	position of a material point in the reference configuration
χ(**X**, *t*)	placement function
**x**(**X**, *t*)	position of the material point in the actual configuration
**F**(**X**, *t*)	material deformation gradient tensor
**C**(**X**, *t*)	right Cauchy-Green deformation tensor
**E**(**X**, *t*)	Green's strain tensor
**T**(**x**, **a**_0_, *f_s_*)	Cauchy stress tensor
**S**(**C**, **a**_0_, *f_s_*)	second Piola-Kirchhoff stress tensor
**a**_0_(**X**)	referential unit vector in fiber direction
*I*_1_(**C**)	first principal invariant of **C**
*I*_4_(**C**, **a**_0_)	fourth (mixed) invariant of **C**
λ_*f*_(**C**, **a**_0_)	fiber stretch
ℓ_*S*_(λ_*f*_)	sarcomere length
${\dot{\ell }}_{S}$(${\dot{\lambda }}_{f}$)	sarcomere contraction velocity
*B*(*f_s_*, ${\dot{\ell }}_{S}$)	sarcomere-based active stress
γ(*f_s_*, ℓ_*S*_, ${\dot{\ell }}_{S}$)	normalized sarcomere-based active stress
Ξ(ℓ_*S*_)	active force-length relation
*x*_1_(*f_s_*, ${\dot{\ell }}_{S}$, *t*)	average distortion of XBs in the *A*_1_ state
*x*_2_(*f_s_*, ${\dot{\ell }}_{S}$, *t*)	average distortion of XBs in the *A*_2_ state
[*D*_2_](*f_s_*, *t*)	concentration of XBs in the detached state
[*A*_1_](*f_s_*, *t*)	concentration of XBs in the attached pre-power stroke state
[*A*_2_](*f_s_*, *t*)	concentration of XBs in the attached post-power stroke state
*f*_0_([*A*_1_], [*A*_2_], *x*_1_, *x*_2_)	XB-attachment rate
*g*_0_(*x*_2_)	XB-detachment rate (from *A*_2_)
*V_m_*(*f_s_*, *t*)	membrane voltage
**y**(*f_s_*, *t*)	state variables of the biophysical half-sarcomere model
*I_ion_*(*t*, *V_m_*, **y**)	ionic currents crossing the cell membrane

**Table 2:** **Model parameters**.

**Symbol**	**Description**	**Value (slow/fast)**	**References**
*c*_10_	1st Mooney-Rivlin parameter (fitted)	6.352e^−10^ kPa	[*A*]
*c*_01_	2nd Mooney-Rivlin parameter (fitted)	3.627 kPa	[*A*]
*b*_1_	1st anisotropy parameter (fitted)	2.756e^−5^ kPa	[*B*]
*d*_1_	2nd anisotropy parameter (fitted)	43.373 [–]	[*B*]
*P^max^*	max. isometric stress	73.0 kPa	[*B*]
*f_s_*	stimulation frequency	single twitch, 50 Hz, 100 Hz	[*C*]
*x*_0_	average distortion induced through the power stroke	0.05 μm	[*C*]
*f*′	XB-detachment rate (from *A*_1_)	5/15 ms^−1^	[*C*]
*h*_0_	power stroke forward rate	0.08/0.24 ms^−1^	[*C*]
*h*′	power stroke backward rate	0.06/0.18 ms^−1^	[*C*]
*f*	XB-attachment rate of an isometric contraction	0.5/1.5 ms^−1^	[*C*]
*g*	XB-detachment rate if no neighbor is in the *A*_2_ state	0.04/0.12 ms^−1^	[*C*]
*T_tot_*	number of possible XB connections at ℓ_*S*_	140 μM	[*C*]
ν	influence of cooperative effects (fitted)	3.0/3.4 [–]	[*D*]
ϑ	level of distortion dependence (fitted)	1700/1000 [–]	[*D*]
ℓ^0^_*S*_	resting sarcomere length	2.0 μm	[*E*]
ℓ^*opt*^_*S*_	optimal sarcomere length	2.4 μm	[*F*]
*C_m_*	membrane capacitance	0.58/1.0 μF/cm^2^	[*C*]
*A_m_*	surface-area-to-volume ratio	500 cm^−1^	[*G*]
σ	conductivity	3.828 mS/cm	[*G*]

*References, [*A*] – Zheng et al. (1999); [*B*] – Hawkins and Bey (1994); [*C*] – Shorten et al. (2007); [*D*] – Ranatunga (1984); [*E*] – Edman (1979); [*F*] – Burkholder and Lieber (2001); [*G*] – Röhrle et al. (2012). The table also indicates which parameters were obtained through fitting*.

### 2.2. The continuum-mechanical muscle model

Since the physiological working range of many muscles involves changes in length of 50% and more (Burkholder and Lieber, 2001), a continuum-mechanical analysis must be based on the finite elasticity theory (Holzapfel, 2000; Bonet and Wood, 2008). In continuum mechanics, the placement function χ assigns a material point with position **X** in the reference (undeformed) configuration at time *t*_0_ to a position in the actual (deformed) configuration **x** at time *t*, i. e., **x** = **χ**(**X**, *t*). The material deformation gradient tensor **F** is defined as the derivative of the placement function with respect to the material coordinates, i. e., $F=\frac{\partial \chi }{\partial X}=\frac{\partial x}{\partial X}$. Local deformations and strains are conveniently described by the right Cauchy-Green deformation tensor **C** = **F**^*T*^**F** and the Green's strain tensor $E=\frac{1}{2}(C-I)$, respectively, where **I** is the second-order identity tensor.

Considering the stress equilibrium in the actual configuration and neglecting inertia and body forces, the momentum balance equation reduces to div **T** = **0**, where **T** denotes the Cauchy stress tensor. To characterize the material behavior, a constitutive equation is required that relates the local deformations or strains to the resulting local stresses. This is conveniently done in the reference configuration. The Cauchy stress tensor of the actual configuration is related to the second Piola-Kirchhoff stress tensor, **S**, of the reference configuration via a scaled covariant push forward operation: **T** = (det **F**)^−1^ **F S F**^*T*^. Muscle tissue can actively generate tension and in the passive state, it exhibits transversal isotropic material behavior. This is reflected in **S**, which consists of an isotropic part based on the Mooney-Rivlin material, **S**^*iso*^, a term appealing to stretches in the fiber direction, **S**^*ani*^ (cf. Markert et al., 2005), which together with **S**^*iso*^ characterizes the transversal isotropic passive behavior of muscle tissue, and a term representing the muscle's ability to actively generate tension, **S**^*act*^. The form of **S** is derived in Heidlauf and Röhrle (2013), and is given by

(1) $$\begin{array}{l} S=S^{iso}+S^{ani}+S^{act}-pC^{-1},\\ S^{iso}=2c_{10}I+2c_{01}(I_{1}I-C),\\ S^{ani}=b_{1}(\lambda_{f}^{d_{1}-2}-\lambda_{f}^{-2})a_{0}⊗a_{0},\\ S^{act}=\lambda_{f}^{-1}P^{act}a_{0}⊗a_{0}, \end{array}$$

where *p* is the hydrostatic pressure, *I*_1_ = tr **C** is the first principal invariant of **C**, and **a**_0_ is a unit vector in fiber direction defined in the reference configuration. Further, $\lambda_{f}=\sqrt{I_{4}}$ denotes the fiber stretch with *I*_4_ = **a**_0_ · **Ca**_0_ being the fourth (mixed) invariant of **C**.

While the fiber stretch is a (spatially varying) macroscopic quantity, it can be related to the corresponding quantity on the sarcomere level, i. e., the sarcomere length, ℓ_*S*_, by λ_*f*_ = ℓ_*S*_/ℓ^0^_*S*_ with ℓ^0^_*S*_ = 2.0 μm being the sarcomere resting length. Finally, *P^act^* represents a scalar-valued active nominal stress, which is the product of the maximum active stress at optimal fiber length and under isometric conditions, *P^max^*, and the normalized active stress *γ*:

(2) $$P^{act}=P^{max}\bar{\gamma }(f_{s},\lambda_{f},{\dot{\lambda }}_{f}).$$

Therein, *γ* depends on the stimulation frequency *f_s_*, the fiber length (represented through the fiber stretch λ_*f*_), and the contraction velocity, ${\dot{\lambda }}_{f}$. Note that previous models (Johansson et al., 2000; Röhrle et al., 2008; Heidlauf and Röhrle, 2013) employ the *F*-*ℓ* and/or *F*-*v* relations on the macroscopic continuum level in the form *P^act^* = *P*^*max*^ *f*_1_(α) *f*_2_(λ_*f*_) *f*_3_(${\dot{\lambda }}_{f}$) with α ϵ [0, 1] being an internal activation parameter. In contrast to these models, the present work provides novel contributions to the field of multiscale skeletal muscle modeling by determining *γ* as part of the biophysical model on the microscale (see next section).

The macroscopic material parameters *c*_10_ and *c*_01_ in Equation (2) have been fitted in a least-squares sense to the uniaxial compression experiments of Zheng et al. (1999). Further, *b*_1_ and *d*_1_ have been determined similarly from the passive experimental data of Hawkins and Bey (1994), from which also the value of *P^max^* in Equation (2) is adopted. The parameters are summarized in Table 2.

### 2.3. The biophysical half-sarcomere model

The basis for modeling the subcellular level in this contribution is the model of Shorten et al. (2007), which describes the complex, nonlinear, biophysical processes leading from electrical excitation to contraction and force generation. To model the excitation-contraction coupling, Shorten et al. (2007) combined several component models describing (a) membrane electrophysiology, (b) calcium release from the sarcoplasmic reticulum and (c) calcium dynamics, (d) cross-bridge (XB) dynamics, and (e) fatigue. The model of Shorten et al. (2007) can be freely accessed and downloaded from the CellML website (http://www.cellml.org/).

**Figure 1:**
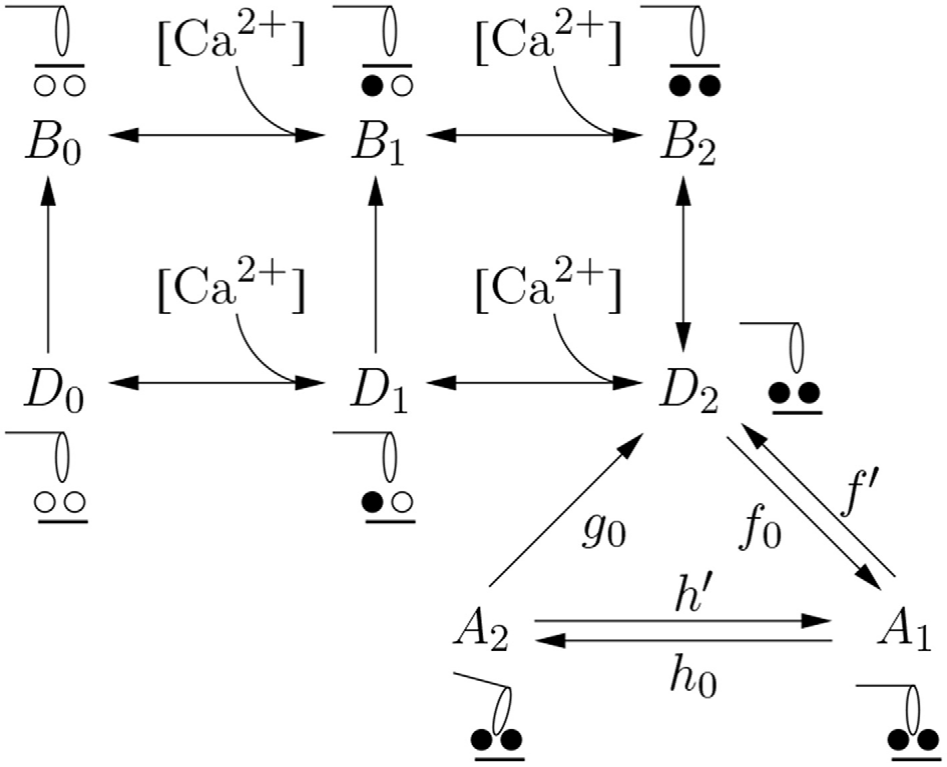
**The cross-bridge dynamics model**. The reader is referred to the text for explanations.

The modifications of this contribution to the model of Shorten et al. (2007) are restricted to the eight-state XB-dynamics component model (d), which is based on the four-state XB-dynamics model of Razumova et al. (1999, 2000) and Campbell et al. (2001a,b). A schematic representation of the eight-state model is shown in Figure 1. In six of the eight states, the XBs are in a detached state with zero, one or two Ca^2+^ ions bound to troponin (denoted by indices 0, 1, and 2, respectively) and with tropomyosin in either a blocking (*B*) or non-blocking (*D*) position. Only in the case when two Ca^2+^ ions are bound to troponin and the tropomyosin block is in a non-blocking position (the *D*_2_ state), the detached XB can move to a state where the myosin head is attached. Two attached states are distinguished—the pre-power stroke state *A*_1_ and the post-power stroke state *A*_2_. The transition from the *A*_1_ to the *A*_2_ state represents the power stroke, i. e., the force producing step, for which the forward and backward reaction rates, *h*_0_ and *h*′, respectively, apply. The forward and backward reaction rates changing the *D*_2_ to the *A*_1_ state and vice versa (XB attachment and detachment) are denoted by *f*_0_ and *f*′, respectively. Finally, the detachment of XBs from state *A*_2_ to state *D*_2_ is described by reaction rate *g*_0_, see Figure 1. Shorten et al. (2007) provide a slow-twitch (type-I) and a fast-twitch (type-II) parametrization of the model to simulate isometric contractions of mouse soleus and EDL muscle, respectively. These parameter sets are adopted in the present work, i. e., no reparametrization is required.

In the present contribution, the model of Shorten et al. (2007) is extended to non-isometric conditions. This is done, first, by incorporating changes in the myofilament overlap, and further, by adding a distortion dependence and cooperative effects to the XB dynamics component model. These extensions are based on the works of Razumova et al. (1999) and Campbell et al. (2001b).

**Figure 2:**
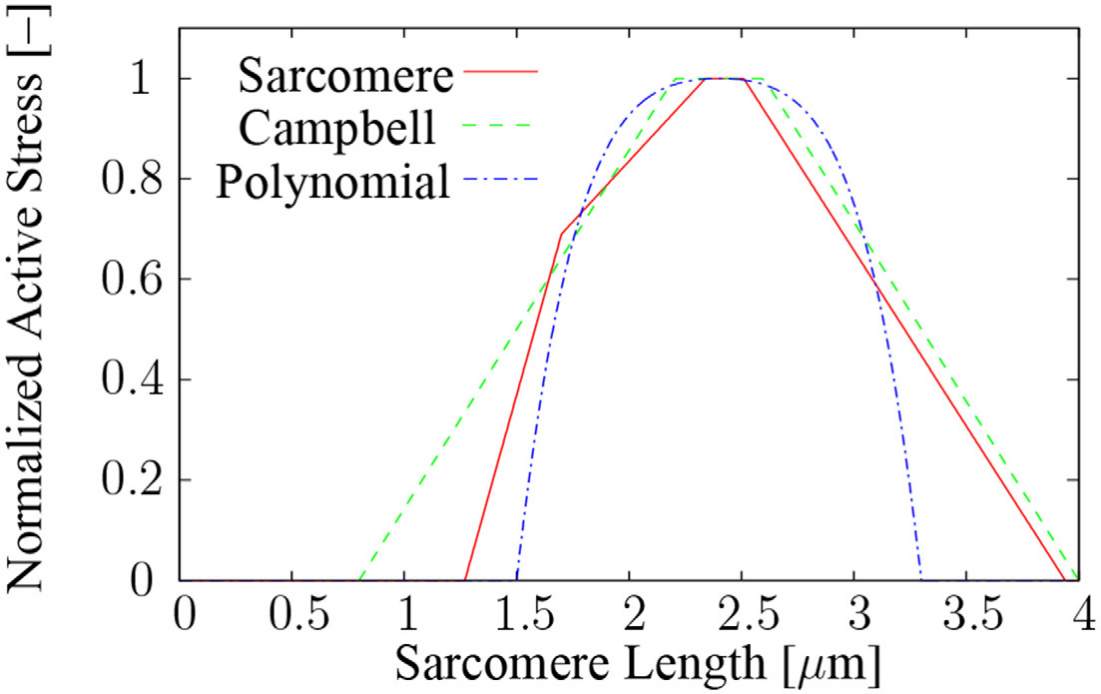
**The relation between the normalized maximum isometric active stress and the sarcomere length**. Plotted is the experimentally determined force-sarcomere length relation for cat skeletal muscle (Rassier et al., 1999) (red solid line), the piecewise linear relation of Campbell et al. (2001b) (dashed green line), and the fourth-order polynomial of this work (dot-dashed blue line).

The force that can be exerted by a sarcomere depends on the number of XB connections between the actin and myosin filaments (Huxley, 1957). The number of possible XB connections depends on the filament overlap, and hence, on the sarcomere length (Gordon et al., 1966). Based on analytical considerations of the filament overlap, Campbell et al. (2001b) proposed a piecewise linear relation between the sarcomere half-length and the number of possible XB connections. The relation is depicted in Figure 2 (green dashed line) assuming a direct relation between the number of possible XB connections and the isometric active force at full activation. Experiments on single sarcomeres, however, suggest a steeper decline of the force on the ascending limb of the active force-length curve at sarcomere lengths below 1.7 μm, and no active force production at lengths below 1.27 μm (Gordon et al., 1966). This is attributed to an interaction of the myosin filament with the Z-disks at low sarcomere lengths. The red solid line in Figure 2 shows the experimentally determined relation between the sarcomere length and the isometric active stress at full activation. In the present work, a fourth-order polynomial is used, cf. Figure 2 (dot-dashed blue line):

(3) $$\begin{array}{l} \Xi (\ell_{S})=\text{max}[-1.2\ell_{S}{}^{4}+11.5\ell_{S}{}^{3}-41.7\ell_{S}{}^{2}+67.6\ell_{S}\\ \;-40.3;\;0], \end{array}$$

where Ξ is the normalized isometric active force at full activation, and ℓ_*S*_ denotes the sarcomere length. The polynomial in (3) is symmetric with respect to the optimal sarcomere length ℓ^*opt*^_*S*_ = 2.4 μm (Burkholder and Lieber, 2001), and can be seen as an approximation to the experimentally determined force-sarcomere length relation, where the largest deviations occur at very long sarcomere lengths. In this contribution, the behavior at very long sarcomere lengths, however, is dominated by the passive stiffness of the muscle tissue, and therefore, the implications of the deviations will be limited. Note that the fourth-order polynomial in (3) is a generic description of a muscle's *F*-*ℓ* behavior (cf. Zuurbier et al., 1995). This approximation can be easily replaced by a different *F*-*ℓ* curve that was fitted to experimental data of a specific muscle. Furthermore, the optimal sarcomere length, which is invariant for all presented simulations, can be changed to simulate a specific muscle.

**Figure 3:**
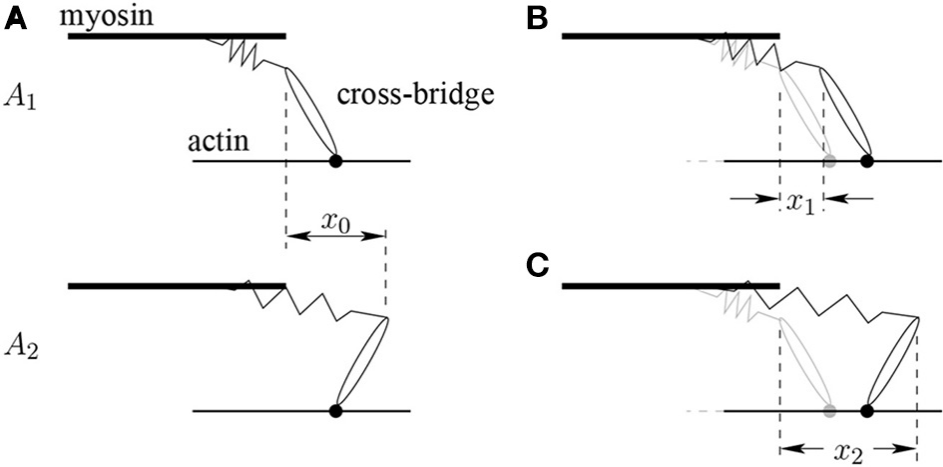
**(A)** The average distortion *x*_0_ induced through the power stroke in an isometric contraction. In the pre-power stroke state *A*_1_ the cross-bridge is attached to the myosin binding site (small filled circle) and does not experience an elastic distortion. The power stroke converts the *A*_1_ to the *A*_2_ state by transducing chemically stored energy into mechanical energy, which is stored in the elastically distorted cross-bridges. **(B)** Average distortion *x*_1_ induced through filament sliding during non-isometric contractions on the cross-bridges in the *A*_1_ state. **(C)** Average distortion *x*_2_ induced through filament sliding during non-isometric contractions on the cross-bridges in the *A*_2_ state.

To account for length changes during contraction, average distortions (or elastic deformations) of XBs in a sarcomere are introduced into the XB-dynamics component model according to Campbell et al. (2001b). The average distortion induced by the power stroke during an isometric contraction of a half-sarcomere is denoted by *x*_0_. The average elastic deformations among XBs in the pre-power stroke state *A*_1_ and post-power stroke state *A*_2_ induced through filament sliding during non-isometric contractions are denoted by *x*_1_ and *x*_2_, respectively. Note that the term average refers in this context to the spatial average over all XBs of that sarcomere in the respective state. Figure 3 illustrates the different distortions. While *x*_0_ is assumed to be constant, *x*_1_ and *x*_2_ account for distortions entering and leaving due to XB cycling and for distortions imposed by shearing between thick and thin filaments. From the distortional balances, Campbell et al. (2001b) derived the following ODEs, which are included in the present model:

(4) $$\begin{array}{l} \frac{\partial x_{1}}{\partial t}=-\left(f_{0}\frac{\left[D_{2}\right]}{\left[A_{1}\right]}+h^{\prime }\frac{\left[A_{2}\right]}{\left[A_{1}\right]}\right)x_{1}+h^{\prime }\frac{\left[A_{2}\right]}{\left[A_{1}\right]}(x_{2}-x_{0})+\frac{{\dot{\ell }}_{S}}{2},\\ \frac{\partial x_{2}}{\partial t}=-h_{0}\frac{\left[A_{1}\right]}{\left[A_{2}\right]}\left(x_{2}-(x_{1}+x_{0})\right)+\frac{{\dot{\ell }}_{S}}{2}. \end{array}$$

Therein, ${\dot{\ell }}_{S}$ denotes the sarcomere contraction velocity. Further, quantities in square brackets denote concentrations of XBs in the respective state. The differential equations describing the concentrations of XBs in the different states are part of the biophysical half-sarcomere model of Shorten et al. (2007).

The force exerted by a half-sarcomere is proportional to the product of the stiffness of all parallel XBs and their average distortions (Razumova et al., 1999), i. e.,

(5) $$B(f_{s},\;{\dot{\ell }}_{S})=[A_{1}]x_{1}+[A_{2}]x_{2}.$$

The normalized sarcomere-based active stress γ is defined to be the product of the force-length relation and the ratio between *B* and the value of *B* at maximum stimulation *f^max^_s_* and under isometric conditions ${\dot{\ell }}_{S}$, corrected for the value of *B* at zero activation:

(6) $$\gamma =\Xi (\ell_{S})\frac{B(f_{s},\;{\dot{\ell }}_{S})-B(0,\;0)}{B(f_{s}^{max},\;0)-B(0,\;0)}.$$

Before, however, including γ in the macroscopic continuum-mechanical constitutive equation (2), the normalized sarcomere-based active stress values are homogenized, cf. Section 2.5 and Heidlauf and Röhrle (2013) for details.

To reproduce the hyperbolic *F*-*v* relation (Hill, 1938), Razumova et al. (1999) proposed two modifications to their four-state XB-dynamics model: (i) The forward rate of XB attachment *f*_0_ contains now nearest-neighbor cooperative effects, i. e., increased XB-attachment probabilities due to neighboring XBs in the force-bearing state. (ii) A distortion dependence is now incorporated in the XB-detachment rate *g*_0_ accounting for an increasing probability of XB detachment with an increasing XB distortion:

(7) $$\begin{array}{l} f_{0}=\bar{f}(1+\frac{\left[A_{1}\right]}{T_{tot}}\left[\exp\left(\frac{x_{1}}{x_{0}}(\nu -1)\right)-1\right]\\ \;+\;\frac{\left[A_{2}\right]}{T_{tot}}\left[\exp\left(\frac{x_{2}}{x_{0}}(\nu -1)\right)-1\right]), \end{array}$$

(8) $$g_{0}=\bar{g}\exp\left(\vartheta {\left(x_{2}-x_{0}\right)}^{2}\right),$$

where *g* is the XB-detachment rate of an isometric contraction, ϑ controls the distortion dependence, *f* denotes the forward rate of XB attachment if no neighbor is in the force-bearing state, and ν controls the influence of the cooperative effects. Further, *T_tot_* is the total number of possible XB connections at optimal filament overlap. Equations (7) and (8) are added to the XB-dynamics component model within the model of Shorten et al. (2007). Note that in the method of Campbell et al. (2001b), *T_tot_* depends on the sarcomere length. This approach, however, bears some problems, for example, in the case when sarcomeres are stretched to beyond myofilament overlap (*T_tot_* → 0). Therefore, the present approach includes the *F*-*ℓ* relation in Equation (6) in a form that is inspired by Hill-type models (Siebert et al., 2008). However, in contrast to Hill-type models that include the *F*-*ℓ* relation at the macroscopic whole muscle level, the present approach contains this relation at the microscopic sarcomere level.

### 2.4. Action potential propagation

Previous electro-mechanical muscle models (Fernandez et al., 2005; Böl et al., 2011) describe the AP propagation as a continuous 3D wave front moving through the entire muscle domain. However, the macroscopic electrical conductivity of skeletal muscle tissue perpendicular to the fiber direction is up to one magnitude lower than the conductivity along the fiber direction (Epstein and Foster, 1983; Gielen et al., 1984), and electrical stimulation from one fiber to adjacent ones is not observed. Therefore, the propagation of an AP along a skeletal muscle fiber is modeled as a one-dimensional (1D) problem (cf. Röhrle et al., 2008; Heidlauf and Röhrle, 2013). The AP propagation can be described by the monodomain equation, which is in 1D identical to the cable equation, see e. g., Hodgkin and Huxley (1952); Pullan et al. (2005):

(9) $$\frac{\partial }{\partial s}(\sigma \frac{\partial V_{m}}{\partial s})=A_{m}(C_{m}\frac{\partial V_{m}}{\partial t}+I_{ion}).$$

Therein, *s* is the spatial variable describing the position along the path of the fiber, σ denotes the conductivity, *V_m_* represents the membrane voltage, *A_m_* reflects the surface-area-to-volume ratio of the cell, and *C_m_* is the capacitance of the cell membrane per unit area. The monodomain equation links through the ionic currents crossing the cell membrane, *I_ion_*, to the half-sarcomere model described in the previous section, i. e., *I_ion_* = *I_ion_*(*t*, *V_m_*, **y**) with **y** denoting the state variables of the half-sarcomere model. The term on the left-hand side of Equation (9) describes the diffusion of membrane potential along a muscle fiber. For details, the reader is referred to Heidlauf and Röhrle (2013).

### 2.5. Computational framework

Due to interactions between the half-sarcomere model, the AP propagation model, and the continuum-mechanical model, a fully coupled system needs to be solved in an integrated fashion. In this contribution, a staggered solution scheme is employed (Heidlauf and Röhrle, 2013), which allows usage of different solution methods and different time step sizes for the solution of the individual subsystems. Moreover, due to computational efficiency, an approach that uses different finite element discretizations for the 1D bioelectrical and the 3D continuum-mechanical subsystems is adopted. Since different meshes are used for different subsystems, transfer operations for sharing variables between different meshes are required. For example, the normalized active stress γ determined in the half-sarcomere models, cf. Equation (6), needs to be homogenized to the coarser 3D continuum-mechanical mesh (Γ: γ → *γ*) to be included in the evaluation of the stress tensor in Equations (1) and (2). Without loss of generality, a geometrically based homogenization is used in this contribution. The convergence behavior of this approach is investigated in Röhrle et al. (2008), and shows good results. Further details on the computational framework can be found in Heidlauf and Röhrle (2013) and Bradley et al. (2011).

## 3. Results

Before comparing series-fibered and spanning-fibered muscles, the behavior of the extended half-sarcomere model and the new fully coupled chemo-electro-mechanical skeletal muscle model is investigated.

### 3.1. Half-sarcomere model

To show that the extended half-sarcomere model (Shorten et al., 2007) exhibits a *F*-*v* relation as muscle fibers do, the sensitivity of the model to the newly introduced parameters ν and ϑ is analyzed first. To do so, experiments are carried out using a single extended half-sarcomere model at a stimulation frequency of *f_s_* = 100 Hz. For different prescribed constant velocities, the corresponding normalized active stresses γ are computed at optimal sarcomere length.

**Figure 4:**
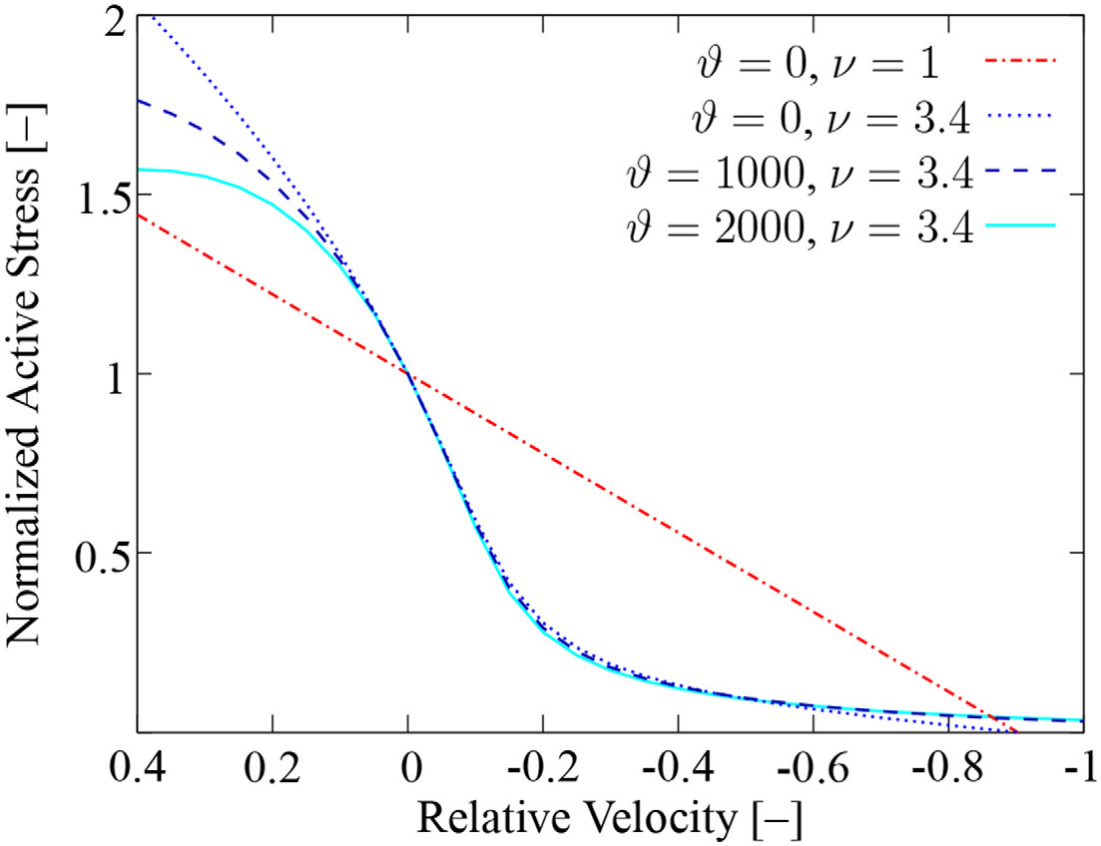
***F*-*v* relation of an isolated half-sarcomere model**. Shown is the relation for constant rate coefficients (red dash-dotted line), for variability in *f*_0_ only (blue dotted line), and for additional variability in *g*_0_ (blue dashed line and turquoise solid line). To depict the Hill relation in its typical form, the x-axis is inverted to show shortening contractions on the right.

The model predicts a linear *F*-*v* relation for constant rate coefficients *f*_0_ and *g*_0_ (ϑ = 0, ν = 1), cf. Figure 4. When considering nearest-neighbor cooperative effects in *f*_0_ (ϑ = 0, ν = 3.4), the model is able to predict a hyperbolic relation for shortening contractions, but unreasonable high forces occur for lengthening contractions. The distortion dependence in *g*_0_ (ν = 3.4, ϑ = 1000, 2000) mainly influences lengthening contractions. Note that Figure 4 only depicts results for the type-II parametrization (Shorten et al., 2007). Similar results are obtained for type-I fibers as demonstrated in Section 3.2.

Further, three shortening contractions are simulated to demonstrate the influence of the *F*-*ℓ* and the *F*-*v* relations on the active stress profiles. To this end, a single half-sarcomere model is stimulated at a frequency of 100 Hz. After 500 ms of isometric contraction at optimal sarcomere length, the sarcomere shortens at a constant prescribed velocity. Three different velocities are considered: 5, 10, and 15% of the maximum shortening velocity *v_max_*.

**Figure 5:**
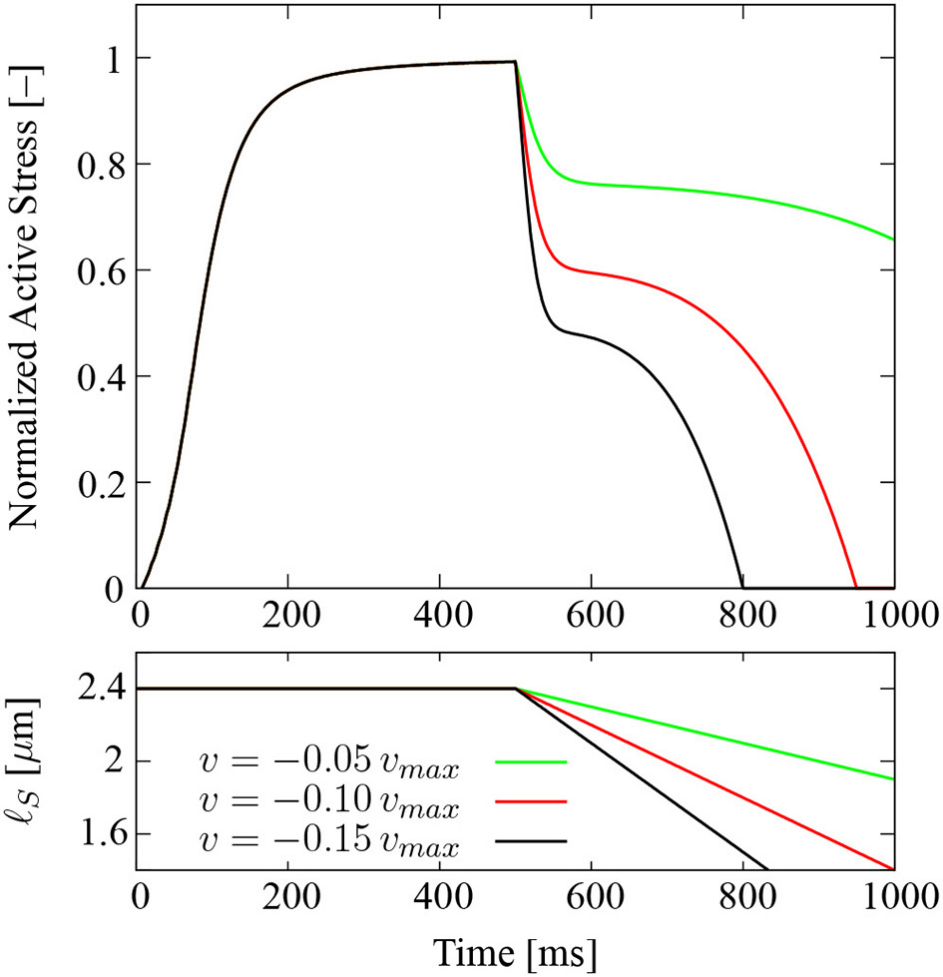
**Evolution of the normalized sarcomere-based active stress of an isolated half-sarcomere model for three different shortening velocities at a stimulation frequency of 100 Hz (top)**. The shortening contraction is preceded by an isometric contraction at optimal length of 500 ms duration. Additionally, the actual sarcomere length (ℓ_*S*_) is shown for each of the stress profiles (bottom).

Figure 5 shows the evolution of the normalized sarcomere-based active stresses (top) and the sarcomere length (ℓ_*S*_, bottom). The profiles show, first, an increase in the active stress due to the stimulation, which is identical for all three traces. After 500 ms, when the active stress approximately saturates and the shortening starts, the model shows an instantaneous stress drop which is due to the shortening velocity. As expected, the magnitude of the stress drop increases with the shortening velocity, cf. Figure 4. The model further predicts a decrease in the stress, which is due to the *F*-*ℓ* relation, i. e., as the sarcomere shortens, it moves along the ascending limb of the *F*-*ℓ* relation (Figure 2) from the optimal length toward smaller sarcomere lengths.

### 3.2. The chemo-electro-mechanical model compared to experimental data

The chemo-electro-mechanical model is first compared to experimental *F*-*ℓ* data to demonstrate that the multiscale muscle model that includes the entire active behavior on the microscale can reproduce typical mechanical behavior of whole muscle on the macroscale. For the comparison, the experimental *F*-*ℓ* data of Hawkins and Bey (1994) are used.

**Figure 6:**
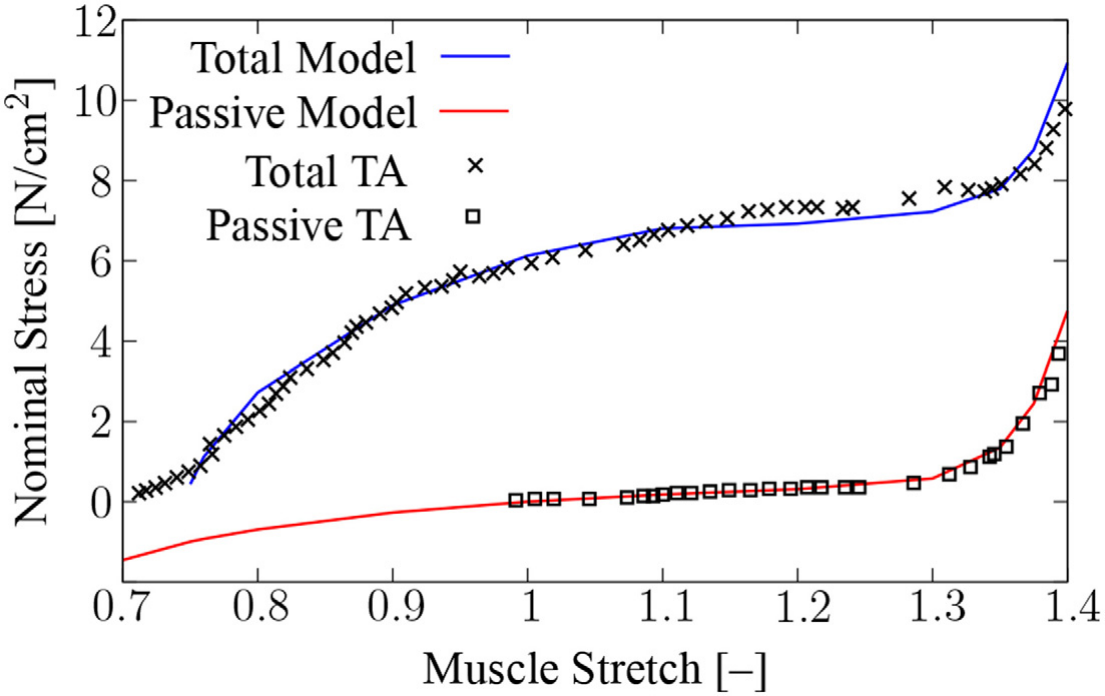
**Muscle stress-stretch relation**. Shown are the passive and total stresses computed using the coupled chemo-electro-mechanical model, and the experimental data of rat TA muscle (Hawkins and Bey, 1994). Simulations are carried out at stretches varying from 0.8 to 1.4 in steps of size 0.1, and at λ_*f*_ = 0.75, 0.76, 1.35, and 1.375.

Hawkins and Bey (1994) analyzed the rat tibialis anterior (TA) muscle, which consists of about 97.5% type-II fibers (Staron et al., 1999). Therefore, in the model all fibers are assumed to be of type II. The numerical specimen used for the comparison is chosen as a rectangular cuboid with dimensions 4 cm × 2 cm × 2 cm. The fibers are aligned with the long edge of the cuboid. Starting from the stress-free reference configuration, the muscle specimen is passively stretched along the fiber direction to the desired muscle length. After passively stretching, displacement in the direction of the fibers is constrained at both ends of the specimen in order to simulate fixed-end contractions. Moreover, displacement at two further non-parallel faces of the specimen is constrained in the direction perpendicular to the respective face (symmetry boundary conditions). Note that the lengths of the individual half-sarcomeres are not constrained but only the total length of the muscle. A stimulation frequency of *f_s_* = 100 Hz is applied to the central half-sarcomere model of each muscle fiber model. The simulation output is the nominal stress, which is defined as the ratio of the resulting reaction forces in fiber direction and the initial cross-sectional area of the specimen. The peak nominal stress of the chemo-electro-mechanical model induced through the passive stretch and the applied stimulation provides the value of the total model. The determined passive and total nominal stresses at different muscle stretches are shown in Figure 6, together with the experimental stress-stretch data of Hawkins and Bey (1994). Note that Hawkins and Bey (1994) used an unrealistic high stimulation frequency of 250 Hz. The biophysical half-sarcomere model can not account for such high frequencies. However, in the model force saturation occurs at a stimulation frequency of about 100 Hz. For this stimulation frequency complete fusion of twitches occurs, which was also reported for the experiment.

After establishing realistic mechanical behavior under isometric conditions, the coupled chemo-electro-mechanical model is now tested for its capacity to reproduce experimental *F*-*v* data of whole muscle. The hyperbolic *F*-*v* relation of Hill (1938) can be expressed by

(10) $$\frac{v}{v_{max}}=\frac{1-F/F_{iso}}{1+F/(\kappa F_{iso})},$$

where κ is a dimensionless parameter, *F_iso_* denotes the maximum isometric force, and *v_max_* is the maximum shortening velocity at *F* = 0.

In the literature, κ ranges from 0.15 to 0.25 (McMahon, 1984). For example, Ranatunga (1984) reports a mean value of κ = 0.24 for rat soleus muscle. Rat soleus muscle consists mainly of type-I fibers (Soukup et al., 2002), and hence, all half-sarcomere models in the chemo-electro-mechanical model use now the type-I parametrization of Shorten et al. (2007). The parameters quantifying the cooperative effects and the distortion dependence are set to ν = 3.0 and ϑ = 1700, respectively.

**Figure 7:**
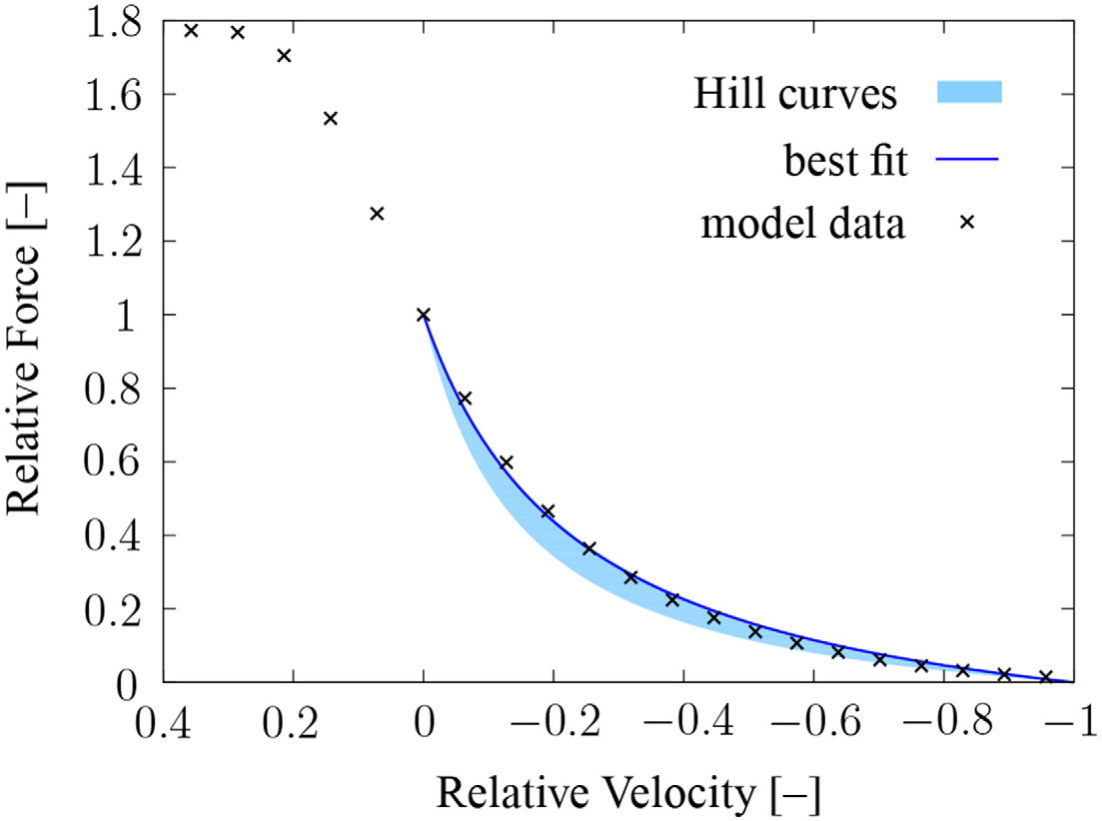
***F*-*v* data computed using the multiscale chemo-electro-mechanical model**. Shown are the *F*-*v* data of the model (black crosses), the corresponding fit of Hill's hyperbolic relation (κ = 0.241, blue line), and the region of typical muscle *F*-*v* curves (0.15≤ κ ≤ 0.25, light-blue shaded area).

Within the numerical experiments, the model specimen is first passively stretched to the optimal length. Then, the length of the specimen is kept fixed, and all fibers are fully activated (*f_s_* = 100 Hz). For a prescribed velocity, the corresponding reaction force is computed. The resulting *F*-*v* data are depicted in Figure 7, where the force values have been normalized to the value at isometric conditions and the velocity has been normalized to the maximum shortening velocity.

Fitting the parameter κ in Equation (10) to the simulation results obtained for shortening contractions in a least-squares sense yields κ = 0.241, cf. Figure 7. For lengthening contractions, the chemo-electro-mechanical model predicts a maximum force of 1.77 times the isometric force, cf. Figure 7.

### 3.3. Compartmentalization

After verifying that the multiscale model is capable of predicting experimental *F*-*ℓ* and *F*-*v* data of whole muscle, the chemo-electro-mechanical skeletal muscle model is used to compare series-fibered and spanning-fibered muscles. The aim of this comparison is to reveal differences in the mechanical behavior of the different muscle fiber arrangements.

In all of the following numerical experiments, a rectangular cuboid with dimensions 12 cm × 2 cm × 2 cm is considered. The fascicle direction is assumed to be aligned with the cuboid's long edge. To mimic series-fibered skeletal muscle arrangements, the long side of the muscle specimen is subdivided into compartments of equal length. The fibers in adjacent compartments are aligned end-to-end, and do not interdigitate with each other. As in real muscle, electrical activation from one fiber to adjacent ones does not occur, neither between adjacent compartments, nor in lateral direction within a compartment. The neuromuscular junction of each fiber is assumed to be located in the middle of the respective fiber. All half-sarcomeres are assumed to be of type II. The mechanical behavior of the chemo-electro-mechanical muscle model is investigated for simultaneously stimulating all fibers. Before stimulating the muscle specimen, it is passively stretched to the optimal length (λ^*opt*^_*f*_ = 1.2, ℓ^*opt*^_*S*_ = 2.4 μm).

**Figure 8:**
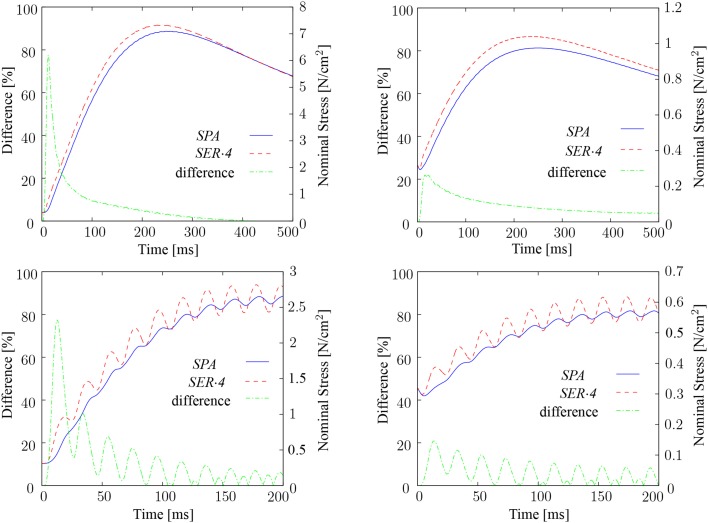
**Comparison of a spanning-fibered muscle model (***SPA***) and a series-fibered muscle model consisting of four in-series arranged fiber compartments (***SER·4***) stimulated with *f_s_* = 100 Hz (top row) and *f_s_* = 50 Hz (bottom row) in fixed-end (left column) and shortening contractions at *v* = 0.1 *v_max_* (right column), and their differences in percent**.

First, fixed-end contractions and shortening contractions at 10% of the maximum shortening velocity at *f_s_* = 50 and 100 Hz are considered. A muscle model with fibers that span the entire length of the fascicles (referred to as *SPA*) and a model consisting of four fiber compartments in series (referred to as *SER*·*4*) are compared to each other. The resulting nominal stresses are depicted in Figure 8. Fixed-end contractions predict differences of almost up to 80% between the different muscle fiber arrangements. The largest differences occur at the beginning of the contraction, i. e., during the first twitch, but decline rapidly to approximately 10% and less. Moreover, the results show that the initial differences are less pronounced in shortening contractions independent of the stimulation frequency. At *f_s_* = 50 Hz, twitches tend to be more fused for model *SPA* than for model *SER*·*4*. This applies to both fixed-end and shortening contractions. Completely fused twitches are observed for both models for *f_s_* = 100 Hz.

Independent of the stimulation frequency, model *SER*·*4* shows higher peak forces than model *SPA* in fixed-end and shortening contractions. At *f_s_* = 100 Hz, the maximum force of model *SER*·*4* is 3.29% and 6.61% higher than the maximum force of model *SPA*, in fixed-end and shortening contractions, respectively. The observed decrease after reaching the maximal value in all simulations with *f_s_* = 100 Hz is due to fatigue, which is contained in the half-sarcomere model of Shorten et al. (2007).

The results reveal that the largest differences between spanning-fibered and series-fibered muscle models occur during the first twitch in fixed-end contractions. Hence, fixed-end single twitch experiments are further investigated in the following. The aim is to reveal a potential relation between the twitch shape and the fiber length.

In addition to the model with spanning fibers (termed *SPA*), muscle specimens consisting of two, four, six, and twelve fiber compartments of equal length are considered. The series-fibered models are termed *SER*·*2*, *SER*·*4*, *SER*·*6*, and *SER*·*12* indicating the respective number of compartments. Furthermore, two different scenarios are considered. In the first scenario, all fibers in all compartments receive a stimulus at the same time to simulate a coordinated single twitch contraction. The second scenario appeals to the model with six in-series arranged compartments, in which only the fibers within the first compartment are stimulated. (Note that the choice which of the compartments is stimulated does not influence the resulting reaction forces.) This model is referred to as *SER*·*6a*.

**Figure 9:**
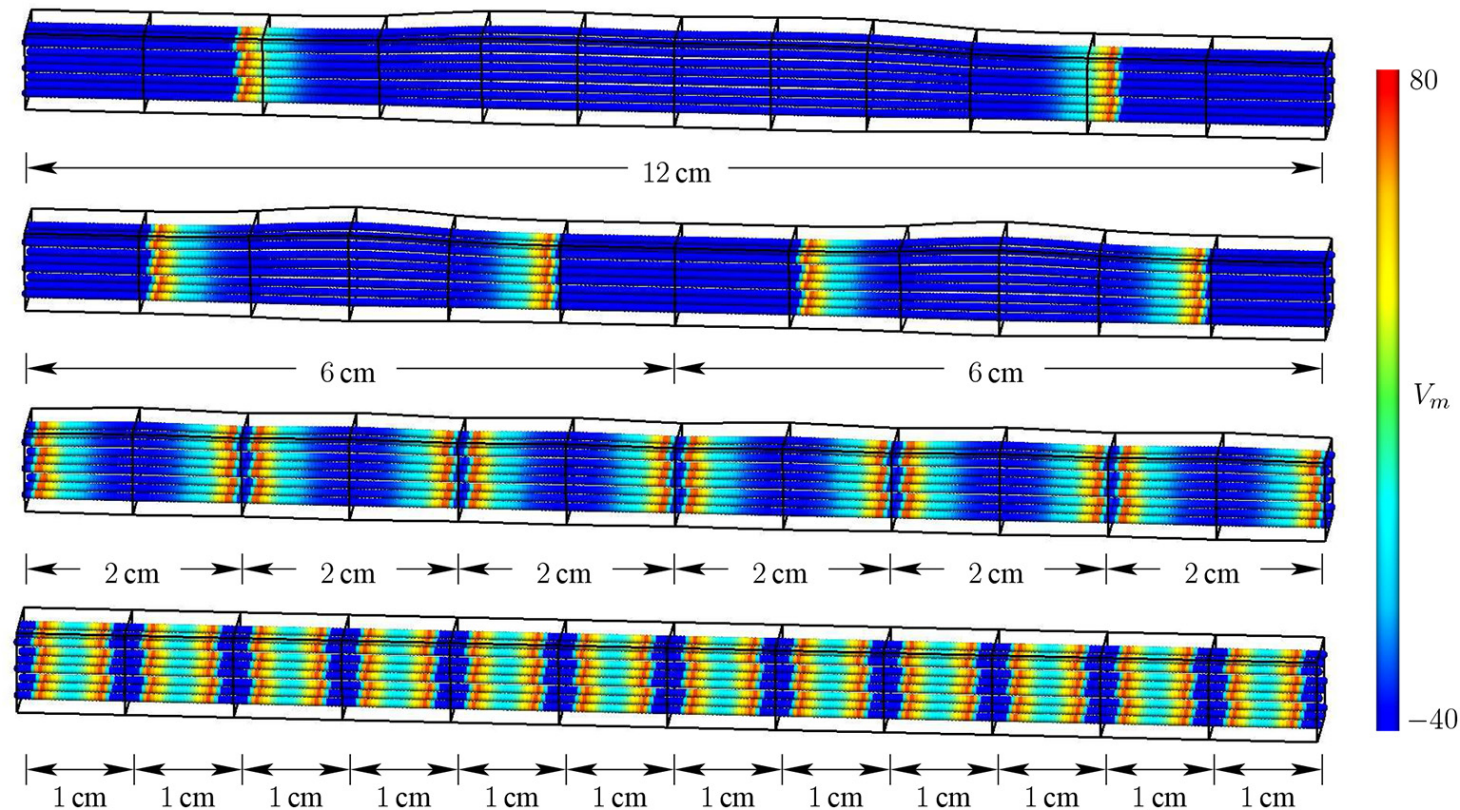
**Distribution of membrane potential, *V_m_* in [mV], and contraction-induced deformation during single twitch contractions of models ***SPA*** (*t* = 22 ms after stimulation), ***SER·2*** (*t* = 10 ms), ***SER·6*** (*t* = 5 ms), and ***SER·12*** (*t* = 2 ms) (from top to bottom)**.

Figure 9 shows the distribution of the membrane potential and the contraction-induced resulting deformation of the muscle in the different models of the first scenario.

**Figure 10:**
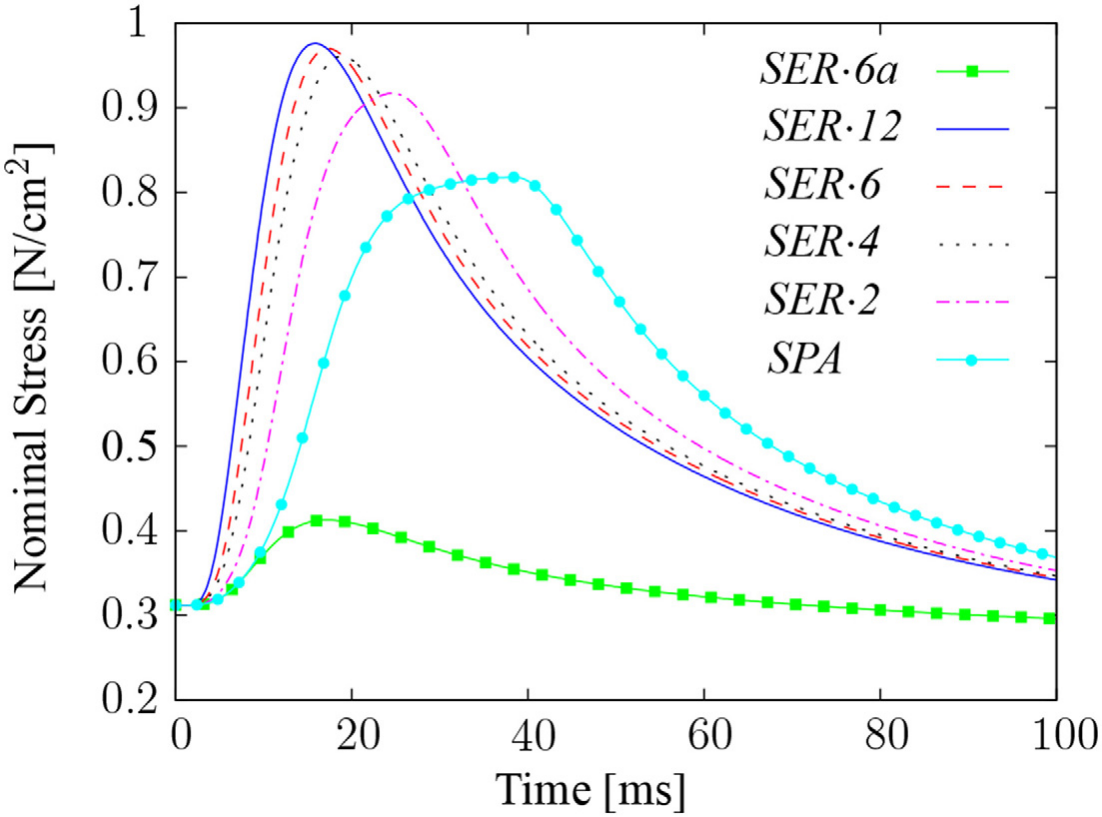
**Comparison of single twitch contractions in a spanning-fibered model and in series-fibered models with different fiber lengths and number of compartments**. The reader is referred to the text for model definitions.

Figure 10 demonstrates that the twitch rise time of a muscle depends on the length of its fibers, i. e., the twitch rise time increases with increasing fiber length. Thus, model *SER*·*12* has the lowest twitch rise time of 17.2 ms, while the maximum twitch rise time occurs in model *SPA*, where the peak stress occurs 38.2 ms after stimulation. The computed AP propagation speed of the models is 2.186 m/s. In model *SPA*, where the AP propagates 6 cm from the motor end-plates to each end of the fibers, this propagation speed yields an AP propagation time of 27.45 ms. In comparison, a half-sarcomere model considered in isolation shows a twitch rise time of 16.1 ms. Hence, the AP propagation time in model *SPA* exceeds the twitch rise time of a single half-sarcomere. In other words, the sarcomeres located at the motor end-plates reach their peak twitch force before the sarcomeres located at the ends of the fibers are activated.

While the twitch rise time increases, the peak twitch stress of the muscle model decreases with increasing fiber length. In detail, the peak twitch stresses are 0.82 and 0.98 N/cm^2^ in models *SPA* and *SER*·*12*, respectively, which corresponds to an increase of 19.4%. Integrating the area below the stress curve over 200 ms, i. e., to a point where the active stress has declined and only passive stress components remain, yields 84.95 N·ms/cm^2^ for model *SPA*, and 83.25 N·ms/cm^2^ for model *SER*·*12*.

Deducting from the total stresses the respective passive stresses, which are due to the initial stretch to optimal length, the peak twitch force obtained in model *SER*·*6a* is 6.5 times smaller than the peak twitch force of model *SER*·*6*.

Further, changes in local sarcomere length during fixed-end single twitch contractions are analyzed. The aim is to investigate if activation-induced stretches of passive sarcomeres to beyond myofilament overlap occur. The resulting maximum and minimum sarcomere lengths are reported in Table 3.

**Table 3:** **Minimum and maximum sarcomere lengths in fixed-end single twitch contractions absolute and in percent of their length prior to stimulation, ℓ^*opt*^_*S*_ = 2.4 μm**.

	**Minimum sarcomere length**		**Maximum sarcomere length**	
*SER*·*12*	2.39 μm	99.59%	2.41 μm	100.41%
*SER*·*6*	2.26 μm	93.96%	2.54 μm	105.95%
*SER*·*4*	2.16 μm	90.05%	2.63 μm	109.68%
*SER*·*2*	2.03 μm	84.52%	2.64 μm	109.84%
*SPA*	1.81 μm	75.49%	2.66 μm	111.02%
*SER*·*6a*	1.74 μm	72.51%	2.58 μm	107.38%

Considering the first scenario, the shortest and largest sarcomere lengths of 1.81 and 2.66 μm, respectively, occur for model *SPA*. Changes in sarcomere length decrease with an increasing number of in-series fiber compartments. In the second scenario, a minimum sarcomere length of 1.74 μm is observed for model *SER*·*6a*.

## 4. Discussion

A multiscale skeletal muscle model was presented that includes the description of the active behavior entirely on the microscopic sarcomere level. Yet, the model proved to be able to reproduce experimentally determined data of whole muscle on the macroscale. The multiscale model was used to investigate differences in the muscle contraction and force generation caused by different muscle fiber arrangements.

### 4.1. From isometric half-sarcomere model to non-isometric whole muscle simulations

The *F*-*ℓ* and *F*-*v* relationships of skeletal muscle originate from properties on the microscopic filament level (Gordon et al., 1966; Piazzesi et al., 2007). For example, Winters et al. (2011) point out that the active *F*-*ℓ* relation of a whole muscle is very similar to the *F*-*ℓ* relation of a single sarcomere. Likewise, the *F*-*v* relation shows very similar characteristics on the cell level (Edman, 1988) and on the whole muscle level *in situ* (Devrome and MacIntosh, 2007). Based on these findings, the proposed model has the advantage to contain the active *F*-*ℓ* and *F*-*v* relations on the microscopic half-sarcomere level.

Extending the half-sarcomere model of Shorten et al. (2007) to non-isometric contractions introduces two more parameters to the model. The additional uncertainty due to the introduction of these parameters is minor, since both of them can easily be determined by comparing computational results to experimental *F*-*v* data. The extended half-sarcomere model can reproduce the hyperbolic *F*-*v* relation of shortening contractions and the bounded force increase in lengthening contractions known from experiments (Hill, 1938; Zajac, 1989). Similar results are reported by Razumova et al. (1999) using a different approach. Razumova et al. (1999) assumed quasi-static conditions and rearranged their XB-dynamics model such that they could compute the corresponding velocity for a prescribed force.

It is noteworthy that, in contrast to previous macroscopic models (Zajac, 1989), the hyperbolic *F*-*v* relation is not explicitly prescribed in the model but results from the XB-dynamics component model formulation. Thus, the model can be used to reveal the underlying mechanisms leading to the characteristic *F*-*v* behavior (Hernández-Gascón et al., 2013).

The active behavior on the macroscopic whole muscle level is modeled to be entirely determined by the extended half-sarcomere model. The presented results demonstrate that the multiscale model is capable of reproducing microscopic properties of the sarcomere level on the macroscopic whole muscle level. This applies likewise to the *F*-*ℓ* and the *F*-*v* relationships.

In the literature, different behaviors are reported for lengthening contractions of skeletal muscles (cf. Morgan, 1990). Zajac (1989) report a bounded increase up to 1.8 times the isometric force, which is adopted in this contribution. Since the model behavior for lengthening contractions proved to be sensitive to a single parameter, the presented model can easily be adapted to a different shape. However, the fact that experimental *F*-*v* relations show a non-continuously differentiable behavior at the transition from shortening to lengthening contractions (Katz, 1939) is not predicted by the model. Once the origin of this unique feature is completely understood, it could potentially be included in the XB-dynamics component model.

### 4.2. Compartmentalization

First the computational results obtained for the different muscle fiber arrangements are discussed, before using this data to analyze its implications on stability.

The presented model predicts the largest differences between series-fibered and spanning-fibered muscles in the rise time, shape and peak force of single twitches. During sustained contractions, twitches tended to fuse at lower stimulation frequencies in spanning-fibered muscles, while series-fibered muscles showed higher peak forces. Since the basic descriptions of passive and active material behavior are identical in the different models, the observed differences in the force responses must result from the differences in the muscle fiber arrangement. Although the same half-sarcomere model is used in all simulations, single twitches are more dispersed in muscle models with longer fibers, which can be explained by longer AP propagation times. Experimentally observed differences in the twitch shape in different fibers of the same twitch type might therefore be largely governed by the fiber length. This might explain the different twitch shapes observed in different species. For example, the twitch rise time in mouse soleus muscle consisting purely of type-I fibers is approximately 35 ms (Shorten et al., 2007), while 90 ms are observed in human type-I motor units (Fuglevand et al., 1993). Further, the simulations demonstrated that a fascicle consisting of end-to-end terminating fibers does functionally not perform like a single muscle fiber of equivalent length, as hypothesized by Lieber and Fridén (2000).

According to Harris et al. (2005), long fibers are less efficient than short fibers since sarcomere shortening cannot be well synchronized along the length of a fiber. Harris et al. (2005) speculate that a twitch in a long fiber will produce much less force than a more synchronous contraction of the sarcomeres. The presented results confirm that the peak twitch force in spanning-fibered muscle is lower than in series-fibered muscle of the same length, however, it is also more dispersed, such that the stress induced through a single twitch integrated over time is similar in series-fibered and spanning-fibered muscles. This can be attributed to the fact that the number of sarcomeres contributing to the active force is identical in both models. The non-activated parts of the fibers behave as series elastic elements, i. e., they store contractile energy. It is believed that the minor differences observed in the integrated stress values stem from local changes in sarcomere length due to the *F*-*ℓ* relation and from different sarcomere contraction velocities due to the *F*-*v* relation. At this point, however, one has to bear in mind that the modeling assumption of hyperelastic passive material behavior neglects viscous effects, which exist in passive muscle (Hoyt et al., 2008; Van Loocke et al., 2008).

The model further predicts that the peak force exerted by a synchronous activation of all in-series arranged compartments exceeds the product of the number of in-series arranged compartments and the peak force produced when stimulating only the fibers in one compartment. This might be explained by the fact that an additional series compliance is introduced through inactive compartments against which the activated fibers contract (Botterman et al., 1983). It is hypothesized that the effect will be more pronounced at shorter muscle lengths than at the optimal length (at which the numerical experiments are carried out) (cf. Mutungi and Ranatunga, 2000), or in muscles with passive forces appearing only at long muscle length (see further below).

Changes in sarcomere length due to the contraction of activated parts of the fibers against non-activated parts are reported for spanning-fibered and series-fibered muscle models. Fixed-end single twitch contractions, in which the fibers of all compartments are simultaneously activated, show that changes in sarcomere length increase with increasing fiber length. Shorter sarcomere lengths are only observed if one out of six compartments is activated (model *SER*·*6a*). This is not surprising as the five non-activated compartments act as series elastic elements. Comparing the extreme values of the sarcomere length with Figure 2 reveals that the range of sarcomere lengths of the numerical experiments is limited to a rather narrow region with considerable filament overlap. Mutungi and Ranatunga (2000) report experimental sarcomere length changes in fixed-end single twitch contractions that are considerably smaller than those found in the present numerical investigations. The difference can be explained based on the fact that Mutungi and Ranatunga (2000) simultaneously stimulated the entire fiber bundle using plate electrodes, and hence, almost all sarcomeres shortened concurrently against a small region at the fiber ends.

The fact that the model predicts rather small changes in sarcomere length during fixed-end single twitch contractions might be explained by the following considerations. A resting sarcomere length of 2.0 μm (Edman, 1979) is assigned to the model's stress-free reference configuration (λ_*f*_ = 1). Thus, the longest observed sarcomere length of 2.66 μm corresponds to a local fiber stretch of λ_*f*_ = 1.33. Comparing this value with the *F*-*ℓ* relation in Figure 6, one observes that considerable passive forces start to appear at this fiber stretch. This can be explained by the fact that at every instant in time, the contractile forces in the activated parts of the muscle need to be matched by the stretch-induced passive forces in the non-activated parts, since they are in-series arranged. Sarcomere length changes will therefore be more pronounced in muscles with passive forces appearing at long whole muscle length.

A description of tendon was not included in the model. Since tendinous tissue is much stiffer than passive muscle tissue (Hawkins and Bey, 1997), the series compliance added to the system by including tendon is small. Therefore, the effect of neglecting tendon in this study is expected to have a minor effect on the force generation and the sarcomere length changes. It should be noted, however, that this only applies to parallel-fibered muscles. In general, tendons and aponeuroses are crucial to muscle-joint dynamics. Therefore, future models should incorporate tendon and aponeurosis compliance to better link sarcomere dynamics to joint dynamics during movement.

The study of compartmentalization is particularly interesting with regard to stability issues. The model results demonstrate that activated parts of a muscle can contract against non-activated parts. It has been hypothesized that in long spanning-fibered muscle, in which the AP propagation time exceeds the twitch rise time, activation-induced stresses might stretch non-activated sarcomeres to beyond myofilament overlap potentially leading to instabilities (Loeb et al., 1987). Loeb et al. (1987) therefore speculate that the twitch rise time might impose a limit on the length of the fibers. The presented results, however, demonstrate that a muscle model, in which the AP propagation time exceeds the twitch rise time of a single sarcomere, does not necessarily show any instabilities. In series-fibered muscle, a similar stability problem is believed to exist when activation of series-arranged compartments is unbalanced or asynchronous, i. e., if fibers in an activated compartment shorten against fibers in non-activated compartments (Richmond et al., 1985; Loeb et al., 1987). This instability was not observed either in the numerical experiments (model *SER*·*6a*) using the presented model settings.

The fact that instabilities are observed neither in the spanning-fibered model nor in the series-fibered model might be due to the fact that in the present model passive forces appear already at short muscle length. According to Hawkins and Bey (1994), this corresponds to the behavior of rat TA muscle, which shows even at full activation a monotonically increasing isometric *F*-*ℓ* relation, cf. Figure 6. The passive stiffness of the muscle tissue might therefore prevent an overextension of non-activated sarcomeres. However, in muscles with passive forces appearing at long muscle length, sarcomere extensions to beyond myofilament overlap might be possible, and this might lead to stability problems and damage (Loeb et al., 1987).

In the future, the proposed multiscale model can be used, for example, to study sarcomere length changes in muscles, in which passive forces appear at long muscle length and the associated potential instabilities. Furthermore, the presented framework can be used to study the implications of the task-specific activation of sub-volumes of a muscle on the muscle contraction and force generation.

### 4.3. Summary

A chemo-electro-mechanical skeletal muscle model has been developed to reveal differences between parallel-fibered muscles, in which the muscle fibers either span the entire length of the fascicles or terminate intrafascicularly. The multiscale model proved to be able to reveal differences in the muscle contraction and force generation that result from the muscle fiber arrangement. The largest differences in the mechanical behaviors due to the different arrangements have been found during fixed-end single twitch contractions. Spanning-fibered muscles showed lower but more dispersed twitch forces than series-fibered muscles of the same length. Similarly, series-fibered muscles showed significantly higher peak forces during sustained contractions. Further, sarcomere length changes during fixed-end single twitch contractions of the multiscale muscle model at optimal sarcomere length have been analyzed. It was found that the sarcomere length changes were limited to a rather narrow region with considerable filament overlap. Stability issues resulting from activation-induced stretches of non-activated sarcomeres to beyond myofilament overlap were not observed. It is concluded that in muscles with passive forces appearing at short muscle length these stability problems do not exist.

### Conflict of interest statement

The authors declare that the research was conducted in the absence of any commercial or financial relationships that could be construed as a potential conflict of interest.
